# β-Glucosidase genes differentially expressed during composting

**DOI:** 10.1186/s13068-020-01813-w

**Published:** 2020-10-19

**Authors:** Xinyue Zhang, Bo Ma, Jiawen Liu, Xiehui Chen, Shanshan Li, Erlie Su, Liyuan Gao, Hongtao Li

**Affiliations:** 1 College of Resources and Environmental Sciences, Northeast Agricultural University, Harbin, 150030 China; 2 School of Animal Medicine, Northeast Agricultural University, Harbin, 150030 China; 3 Northeastern Science Inspection Station, China Ministry of Agriculture Key Laboratory of Animal Pathogen Biology, Harbin, 150030 China

**Keywords:** β-Glucosidase, Differential expression, Functional microbial community, Carbon catabolite repression, Compost

## Abstract

**Background:**

Cellulose degradation by cellulase is brought about by complex communities of interacting microorganisms, which significantly contribute to the cycling of carbon on a global scale. β-Glucosidase (BGL) is the rate-limiting enzyme in the cellulose degradation process. Thus, analyzing the expression of genes involved in cellulose degradation and regulation of BGL gene expression during composting will improve the understanding of the cellulose degradation mechanism. Based on our previous research, we hypothesized that BGL-producing microbial communities differentially regulate the expression of glucose-tolerant BGL and non-glucose-tolerant BGL to adapt to the changes in cellulose degradation conditions.

**Results:**

To confirm this hypothesis, the structure and function of functional microbial communities involved in cellulose degradation were investigated by metatranscriptomics and a DNA library search of the GH1 family of BGLs involved in natural and inoculated composting. Under normal conditions, the group of non-glucose-tolerant BGL genes exhibited higher sensitivity to regulation than the glucose-tolerant BGL genes, which was suppressed during the composting process. Compared with the expression of endoglucanase and exoglucanase, the functional microbial communities exhibited a different transcriptional regulation of BGL genes during the cooling phase of natural composting. BGL-producing microbial communities upregulated the expression of glucose-tolerant BGL under carbon catabolite repression due to the increased glucose concentration, whereas the expression of non-glucose-tolerant BGL was suppressed.

**Conclusion:**

Our results support the hypothesis that the functional microbial communities use multiple strategies of varying effectiveness to regulate the expression of BGL genes to facilitate adaptation to environmental changes.

## Background

Composting is an effective strategy to improve resource utilization, transforming cellulose waste into environmentally friendly end-products by microbial activity. Hence, in-depth knowledge of the succession of a bacterial community is necessary for effective compost management [1, 2]. Cellulose is the most abundant carbohydrate available from plant biomass and can be used as a carbon source during composting. All cellulose-degrading organisms possess cellulase systems comprising multi-enzyme complex of three enzymes: exoglucanase (also designated cellobiohydrolases, EC 3.2.1.91), endoglucanase [EC 3.2.1.4], and β-glucosidase (BGL, EC 3.2.1.21), which act synergistically for the complete hydrolysis of cellulose. BGLs play a pivotal role in this enzymatic system wherein they complete the final step of hydrolysis by converting cellobiose, an intermediate product of cellulose hydrolysis, to glucose. This reaction is the rate-limiting step in the enzymatic hydrolysis of cellulose to glucose [3–5]. Berlemont and Martini [6] reported that BGL genes are present in nearly all bacterial phyla, which was confirmed in Pathan’s research [7]. Hence, a wide range of phylogenetically diverse microorganisms likely participates in the final step of enzymatic cellulose hydrolysis. Therefore, to achieve a high conversion efficiency for cellulose, an improved understanding of the structural and functional contributions made by BGL-producing microbial communities to cellulose degradation during composting is warranted.

β-Glucosidases (BGL, EC 3.2.1.21) are ubiquitous enzymes found in archaea, eubacteria, and eukaryotes [8]. Based on their evolutionary and structural properties, they can be classified into five glycoside hydrolase families (GHs):1, 3, 5, 9, and 30 [8, 9]. The vast majority of isolated bacterial BGLs belong to the GH1 family [8]. Moreover, a high concentration of glucose inhibits BGL activity; however, several BGLs were tolerant to glucose inhibition. To date, many glucose-resistant enzymes have been isolated from bacteria, fungi, and archaea, and identified by metagenomic analyses [3]. For example, rBgl4 from *Talaromyces funiculosus* [10], BGL from *Thermoanaerobacterium aotearoense P8G3#4* [11], G1mgNtBG1 from *Nasutitermes takasagoensis* [12], and BGL6 and Ks5A7 from the metagenomic library [4, 13] have been characterized. Further, all currently identified glucose-tolerant BGLs are members of the GH1 and GH3 families [14], with members of the GH1 family demonstrating superior glucose tolerance [15]. Comparative analysis of the 3D structures of GH1 BGLs reveals the (β/α)_8_ TIM-barrel architecture typical of this enzyme family [16]. De Giuseppe et al. and Santos et al. [15, 17] reported a possible molecular mechanism of glucose tolerance in this enzyme family. They suggested that the bulky side chains W168 and L173 in the Bglhi cause a narrowing of the + 2 aglycone site, thereby restricting access of saccharides to the active site.

However, the “when, why, and how” related to glucose-tolerant BGL regulation by BGL-producing microbial communities in a natural environment have not yet been elucidated. In our previous study, we found opposite tendencies in terms of expression efficiency between two groups of BGL genes under high glucose conditions [18]. Furthermore, additional studies reported that some cellulolytic fungi upregulate glucose-tolerant BGL expression, with added glucose or cellulosic substrates in the culture media [19–21]. We, therefore, hypothesized that BGL-producing microbial communities differentially regulate expression of glucose-tolerant BGL and non-glucose-tolerant BGL genes to adapt to changes in cellulose degradation. However, it is unclear whether and how the mechanism of BGL differential expression in bacteria takes place in complex ecological environments. Thus, in this study, the function and biosynthetic pathways of cellulolytic microbial communities in the thermophilic and cooling phases during natural and inoculated composting were investigated by metatranscriptomics. In addition, we constructed a DNA library for GH1 family bacteria BGL. Of note, Trp168 and Leu173 were highly conserved in glucose-tolerant GH1 enzymes [15]; thus, glucose tolerance or non-tolerance properties of these specific GH1 BGL from DNA library enable to character by sequence similarity data analysis. This study found that the BGL-producing microbial communities differentially expressed BGL genes during composting. Altogether our results may help understanding how microbial populations interact with substrates and products to drive fundamental ecological processes of cellulose degradation.

## Results

### Composting process and metatranscriptome metrics

On average, 45 million raw sequence reads were obtained from the metatranscriptome of each compost, and 27.17 Gbp high-quality sequences were obtained after removing the adapters and quality filtering > 98% of bases with a quality score of at least 30 (Q30). A total of 707,980 unigenes were identified after de novo assembling using Trinity and clustered with CD-HIT. The average length of these unigenes was 425 bp. The average GC content was 49.07%. Rarefaction analysis showed clear asymptotes for four communities, suggesting sufficient sequence coverage to detect the most highly expressed genes.

### Phylogenetic classification of expressed cellulolytic enzymes

**Fig. 1:**
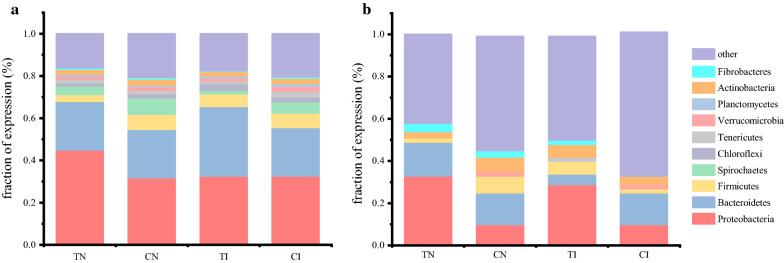
Relative taxonomic distribution of metatranscriptome. **a** Total gene and **b** cellulolytic gene at the phylum level.* TN* thermophilic stage in natural compost,* CN* cooling stage in natural compost,* TI* thermophilic stage in inoculated compost,* CI* cooling phase in the inoculated compost

Total genes and those cellulolytic genes identified by metatranscriptome analysis were phylogenetically classified. At the phylum level, expressed genes predominantly belonged to Proteobacteria, Bacteroidetes, Firmicutes, and Spirochaetes in all samples (Fig. 1a). Notably, Firmicutes, Proteobacteria, Bacteroidetes, and Actinobacteria were also reported as the dominant phyla in other lignocellulosic composts [22, 23]. The expression of the cellulolytic metatranscriptome was similarly dominated by Proteobacteria and Bacteroidetes in all communities (Fig. 1b). As observed previously, Proteobacteria dominates oxygenated habitats [24] while Bacteroidetes are known for their contribution to the largest reservoir of CAZymes in various environments [24, 25]. Among these communities, Actinobacteria, Firmicutes, and Ascomycota expressed a similar, yet minor, fraction of the total cellulolytic metatranscriptome (Fig. 1b). These results agree with those reported by Berlemont et al. [26]. Furthermore, total cellulolytic metatranscriptome analysis revealed that a considerable proportion of the unigenes encoding cellulolytic enzymes were unclassified even at the phylum level (Fig. 1b), which might be attributed to the relatively short genetic sequences or suggest the existence of novel and uncharacterized cellulolytic microbes in the compost.

### Limited cellulose degradation occurs during the cooling phase of natural compost

**Fig. 2:**
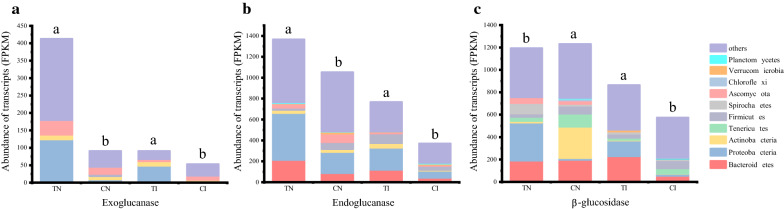
Transcription level of key enzymes in cellulose degradation during composting procedures. Y-axis indicates the share of reads in the total metatranscriptome in FPKM (fragments per kilobase of transcript sequence per millions base pairs sequenced). **a** Exoglucanase transcription. **b** Endoglucanase transcription. **c** β-Glucosidase transcription. Significant differences in read abundances among different composting phases are indicated by different letters (P < 0.05). *TN* thermophilic stage in natural compost, *CN* cooling stage in natural compost, *TI* thermophilic stage in inoculated compost, *CI* cooling phase in inoculated compost

The transcription of genes involved in cellulose degradation, including endoglucanase, exoglucanase, and BGL, were detected in the metatranscriptome (Fig. 2). In both the inoculated and natural compost, the transcription of endoglucanase and exoglucanase genes in the thermophilic phase was significantly higher than that in the cooling phase (*P* < 0.05; Fig. 2a, b). In the inoculated compost, the change in expression of BGL genes showed the same trend as that of endoglucanase and exoglucanase genes, while in the natural compost, the transcription of BGL genes in the cooling phase was significantly higher than that in the thermophilic phase (*P* < 0.05).

Transcriptional regulators, including activators, ClrB and XlnR, and repressors, CreA, play crucial roles in cellulase expression [27, 28]. Compared with the inoculated compost, the expression of activators (ClrB and xlnR) was higher in the thermophilic phase, while the expression of repressors (CreA) was higher in the cooling phase of natural compost (Table 1). Hence, transcriptional regulators exhibited a co-suppression effect on cellulase expression. This result further explained the lower rate of cellulose degradation and decreased CMCase and BGL activity observed during the cooling phase of natural compost in our previous study [18].

**Table 1:** Expression of transcription regulators in both types of composts

	Expression of TN	Expression of CN	Expression of TI	Expression of CI	Source
Activator					
clrb	17.41	10.11	1.46	2.21	Bacteria
xlnR	17.82	3.05	12.17	2.79	Fungi
Repressor					
creA	5.13	33.82	31.58	14.56	Bacteria/fungi

Values represent the number of shared reads in the total metatranscriptome in FPKM (fragments per kilobase of transcript sequence per millions of base pairs sequenced)

*TN* thermophilic stage in natural compost, *CN* cooling stage in natural compost, *TI* thermophilic stage in inoculated compost, *CI* cooling phase in the inoculated compost

**Fig. 3:**
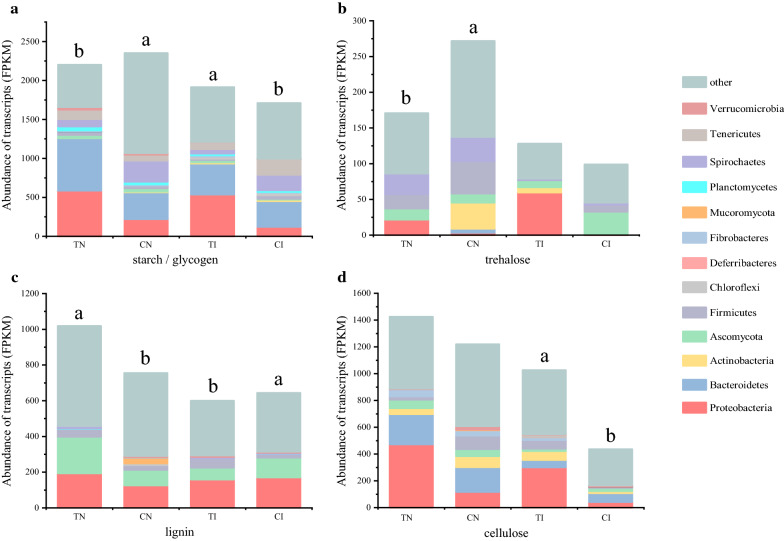
Transcription level of functional groups during composting procedures. Values represent the share of reads in the total metatranscriptome in FPKM (fragments per kilobase of transcript sequence per millions base pairs sequenced). Significant differences in read abundances between the different composting phases are indicated by different letters (P < 0.05). *TN* thermophilic stage in natural compost, *CN* cooling stage in natural compost, *TI* thermophilic stage in inoculated compost, *CI* cooling phase in inoculated compost

Carbon catabolite repression (CCR) has a considerable influence on the selection of microorganisms for carbon sources. The transcript pools differed significantly (*P* < 0.05) between the TN and CN, which likely reflects the difference in microbial carbon source utilization profiles. The microbes in the CN were marked by an increase in the use of simple C compounds (starch and trehalose), while the level of CAZymes targeting recalcitrant plant biomass decreased (Fig. 3). These results indicated that the degradation of complex compounds was inhibited due to CCR.

### Characterization of BGL-producing GH1 families in microbial communities

**Fig. 4:**
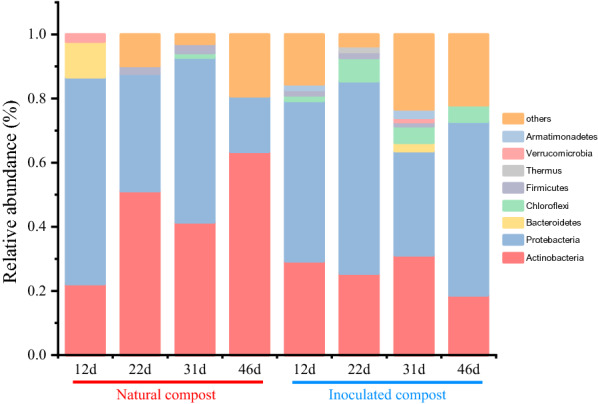
Relative taxonomic distribution of GH1 family bacterial BGL genes from the DNA library during composting procedures. Colors in stack bars indicate taxonomic affiliation of genes. Other sequences belong to bacteria, but their identification at the phylum level is unclear

The designed degenerate primer amplified a gene sequence of approximately 1100–1200 bp in length, including the characteristic sequence, making it possible to identify function-discriminating residues. A total of 457 GH1 family bacterial BGL genes were obtained from the DNA library. The structure of the GH1 BGL-producing bacterial communities was analyzed in both types of compost. Results show that in both composts, Actinobacteria and Proteobacteria contributed to most of the BGL sequences of the GH1 family (Fig. 4), which is consistent with the results reported by Pathan et al. [7] and Zang et al. [29]. Meanwhile, the contribution of Proteobacteria to the thermophilic community was higher than the cooling community, which was confirmed in the metatranscriptome (Fig. 2c). In the natural compost, the abundance of Actinobacteria gradually increased with the composting process, which was not observed in the inoculated compost, as confirmed by metatranscriptome analyses. Actinobacteria play an important role in decomposing organic materials in composts, particularly the degradation of macromolecules such as cellulose, hemicellulose, etc. [30].

### Mechanism of BGL transcriptional regulation

**Fig. 5:**
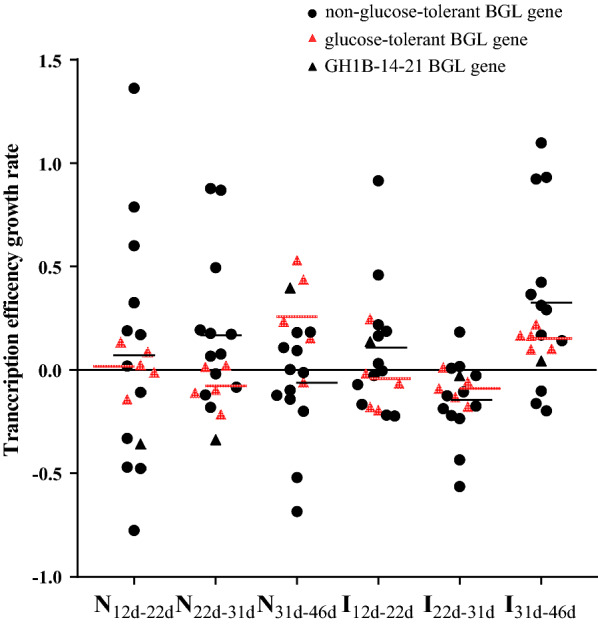
Transcription efficiency growth rate of individual BGL genes during composting procedures. Red triangles represent the individual glucose-tolerant BGL genes. Black circles represent the individual non-glucose-tolerant BGL genes. Black triangles represent the GH1B-14-21 BGL gene. The red line is the average value of transcription efficiency growth rate of the glucose-tolerant BGL gene group, while the black line is the average value of transcription efficiency growth rate of the non-glucose-tolerant BGL gene group

To explore the mechanism underlying the transcriptional regulation of BGL gene expression in the microbial community during the composting process. We selected 18 representative BGL genes from diverse bacterial phyla from the DNA library and successfully quantitatively analyzed these genes from DNA and RNA pools (Additional file 2: Figure S1). Based on the observation that Trp168 and Leu173 were conserved in glucose-tolerant GH1 enzymes using Giuseppe’s method [15], 5 of the 18 BGLs were determined to contain Trp 168 and Leu173 conserved residues, namely GH1B-3-55, GH1B-b4-18, GH1B-7-4-17, GH1B-12-33, and GH1B-13-55, and were regarded as glucose-tolerant BGL genes. The transcriptional regulation of each individual gene could then be determined based on copy numbers DNA and RNA during composting since the abundance of each gene differed between phases. Pathan et al. reported that even BGL genes with low DNA abundance might be functionally important, as revealed by their high expression in some microbes [7]. Hence, we calculated the transcription efficiency (TE) (Additional file 2: Figure S2) and TE growth rate (Fig. 5). Results show that during the cooling phase of natural compost (day 31 to day 46), the average TE growth rate of the non-glucose-tolerant and glucose-tolerant BGL genes reached − 6.1 and 26.0%, respectively, demonstrating that the TE of most non-glucose-tolerant BGL genes were downregulated during this phase. Specifically, the TE growth rate of genes GH1B-b2-20, GH1B-13-2-6, GH1B-b4-37, GH1B-b4-26, GH1B-13-12 and GH1B-10-4-50 reached − 68.4, − 51.9, − 19.9, − 14.0, − 12.1 and − 9.8%, respectively. However, the GH1B-14-21 gene exhibited a TE growth rate that differed compared to the other non-glucose-tolerant genes in that it reached 39.6%, which was comparable to that of the glucose-tolerant genes, which were generally found to be upregulated during the cooling phase. Specifically, the TE growth rate of GH1B-3-55, GH1B-b4-18, GH1B-12-33 and GH1B-13-55 reached the 53.1, 15.4, 43.6 and 23.7%, respectively.

## Discussion

Functioning microbial communities play critical roles in cellulosic biomass breakdown. The study of functional microbes, and how they are associated with, and regulate, cellulose degradation is significant to the cycling of carbon on a global scale. In our previous study, we found that BGL genes are differentially expressed at a glucose concentration of 78.70 mmol kg^−1^ [18]. Differential expression of various BGLs in response to carbon sources supplied in laboratory conditions has been reported previously [31, 32] and likely serves as an adaptation of the microbe to respond to changes in the immediate environments. Hence, we hypothesized that the BGL-producing microbial communities differentially regulate the expression of glucose-tolerant BGL and non-glucose-tolerant BGL to adapt to the changes in cellulose degradation. To verify this hypothesis, the function, and succession of cellulolytic microbial communities, as well as the expression of GH1 family BGL-encoding genes, in cellulose degradation was investigated in natural and inoculated composts. These results showed that inoculating agents caused differences between the two composts, including degradation efficiencies of cellulose, functional microbial activity, and cellulolytic microbial communities. Compared with inoculated compost, CCR occurred when glucose and cellobiose content accumulated in the cooling phase of natural compost.

In both composts, the contribution of Proteobacteria in the thermophilic phase was higher than that in the cooling phase in cellulolytic metatranscriptome (Fig. 1a); this phenomenon was also observed in the total metatranscriptome of natural compost (Fig. 1b). In the inoculated compost, the abundance of species belonging to the phyla Bacteroidetes and Ascomycota increased in the cooling phase. In contrast, the abundance of species belonging to the phyla Actinobacteria and Firmicutes increased in the cooling phase of the natural compost (Fig. 1a). Considering that community structure is closely related to the function of microbial communities [33], these differences in the cooling phase of the two composts may result from the self-adaptation mechanism of functional microbes when they are under carbon-metabolizing pressure, such as degradable cellulose, causing an increase in the concentration of cellobiose and glucose in the natural compost [18].

Compared with the thermophilic phase, the expression of cellulase decreased in the cooling phase of both composts (Fig. 2). Considering that the degree of cellulose degradation and enzyme activity differs between the natural and inoculated composts [18], we speculated that the mechanism by which cellulase expression decreased in the two types of compost differed. Moreover, in the inoculated compost, the expression of exoglucanase, endoglucanase, and BGL was significantly increased during the thermophilic phase compared to the cooling phase (Fig. 2) because the thermophilic phase serves as the main stage of cellulose degradation. Unlike the inoculated compost, the cooling phase of natural compost maintained a higher level of biodegraded cellulose and cellobiose [18], both of which are generally considered cellulase inducers [34]. Therefore, the decreased expression of exoglucanase and endoglucanase genes during the cooling phase was inhibited by high glucose concentration, rather than being induced by cellulose and cellobiose. Unusually, the expression of BGL genes was not inhibited by high glucose concentration, which is caused by the upregulated expression of glucose-tolerant BGL genes under high glucose concentration, as discussed below.

In the cooling phase of natural compost, the presence of sufficient glucose would lead to CCR, which significantly impacts the decomposition of cellulose by functional microorganisms. Cellulase formation occurs in response to the activity of persistent regulators, apparently because of respective consistent regulators. In the study performed by Li et al. [27], the transcriptional regulatory network was described using a “seesaw model” in which the coordinated regulation of cellulolytic genes was established by counteracting activators and repressors. In this study, the expression of cellulase was inhibited by coordinating activity between ClrB, XlnR, and CreA. Microbial carbon utilization profiles indicated that microbes prefer simple carbon sources to cellulose during the cooling phase of natural compost. Meanwhile, the presence of high glucose concentrations, downregulation of endoglucanase and exoglucanase genes, and low activity of CMCase and BGL confirmed that CCR occurred during the cooling phase of the natural compost [18].

The BGL gene expression was also subject to CCR, which functions when a favorable carbon source, such as glucose, is present. Most BGLs are commonly inhibited by glucose [13]. However, in our previous study [18], the expression of certain GH1 family BGL genes was not inhibited by glucose but rather upregulated, leading to the opposite trend, a decrease of enzyme activity. Therefore, based on our results and those of other research teams [19, 20, 35–37], we speculated that functional communities differentially express two types of BGLs in the cooling phase of natural compost. In this study, 5 five glucose-tolerant and 13 non-glucose-tolerant BGL genes were successfully, quantitatively analyzed from DNA and RNA pools. The non-glucose-tolerant BGL genes exhibited more sensitivity to regulation than the glucose-tolerant BGL genes. In addition, the expression of many glucose-tolerant BGL genes was suppressed throughout most of the composting process. In the inoculated compost, regulatory changes in the non-glucose-tolerant BGL gene expression showed the same trend as that of BGL activity, indicating that this group of BGLs were the primary contributors to the BGL activity. Similarly, Mathew et al. showed that increasing BGL production was accompanied by a decrease in the proportion of glucose-tolerant BGL [21], indicating that the expression of non-glucose-tolerant BGL is a common economic microbial behavior. In the natural compost, the non-glucose-tolerant BGL genes were upregulated from 22 to 31 days, which may be related to the increase in cellobiose concentration as a certain concentration of cellobiose can reportedly induce BGL gene expression [34]. As expected, from 31 to 46 days, with the increase in glucose concentration, the expression of non-glucose-tolerant BGL genes was inhibited. However, the expression of glucose-tolerant BGL genes was upregulated compared to that in other phases (Additional file 2: Figure S2). Hence, when the expression of non-glucose-tolerant BGL genes was inhibited, the functional microbial communities upregulated the expression of glucose-tolerant BGL genes under high glucose concentrations. The upregulation of glucose-tolerant BGL genes was related to the increase in Actinobacteria abundance on day 46. We found more than 80% of the GH1 family BGL-producing Actinobacteria microbes contained the Trp 168 and Leu 173 conserved residues of glucose-tolerant BGL (unpublished data). We speculated that the functional microbial community in different niches differentially express BGLs with different biological functions under high glucose concentration. Recently, an analysis of 5,123 annotated bacterial genomes revealed that only 24% were classified as potential degraders (containing genes encoding endoglucanase, exoglucanase, and BGLs), while 56% were potential opportunistic strains (containing genes encoding BGLs but not endoglucanase or exoglucanase) [6]. Hence, a broader phylogenetic diversity of microorganisms expresses BGL than endoglucanase and exoglucanase, which has also been confirmed in a previous study [38]. Hence, it is reasonable to consider that the BGL-producing microbial communities strategies to respond to inhibition caused by high glucose concentration, including adjusting the community structure and differentially expressing various BGLs.

Indeed, the differential expression of BGL in some cellulolytic microbes such as *T. reesei*, *A. oryzae*, *Clostridium thermocellum*, and *Aspergillus terreus* has been reported. The differential expression between cel3a and cel1a regulated in response to glucose concentration was found in *T. reesei*, wherein the expression of cel3a was higher when excess glucose was present than when glucose was depleted from the medium [19]. In the presence of glucose, the expression of other BGL genes was decreased in the Δxyr1 mutant compared to that in the parental strain, except for cel3a [35]. Interestingly, the *T. reesei* strains, TRB1 and SEU-7, exhibited better resistance to CCR for higher expression of cel3a [36, 37]. *A. oryzae* produced two distinct extracellular BGLs: the major form of BGL was highly inhibited by glucose, while the minor form of HGT-BG was highly glucose-tolerant. The expression levels of both were significantly altered when various carbon sources were used in the culture media. Note, BGL exhibited the lowest level of hydrolyzing activity on lactose medium, while a high ratio of HGT-BG was produced by *A. oryzae* [20]. Based on the above results, the functional microbial community uses multiple strategies of varying effectiveness to regulate the expression of BGL genes to adapt to environmental changes during the composting process.

In this study, the GH1 family glucose-tolerant BGL identification method based on highly conserved residues (Trp168 and Leu173) was proposed, consistently with the expected results. However, changes in GH1B-14-21 gene expression regulation showed the same trend as that of glucose-tolerant BGL genes during both types of composting (Fig. 5). Nevertheless, the increased copy number and the overexpression cannot prove that GH1B-14-21 BGL is a glucose-tolerant enzyme. To date, several mechanisms for glucose tolerance of BGL have been reported, including transglycosylation to glucose [39], relief of substrate inhibition [11, 39], and allosteric effects [40, 41]. Of note, certain BGLs have high glucose tolerance despite not containing the Trp168 and Leu 173 residues [42, 43]. Thus, further studies are warranted to reveal the molecular characteristics and transcriptional regulation of the GH1B-14-21 BGL.

## Conclusions

With changes in carbon-metabolizing pressures that occur during composting, the structure and function of BGL-producing microbial communities must also adapt and change. Compared with the glucose-tolerant BGLs, those that were non-glucose-tolerant primarily contributed to BGL activity, exhibiting higher sensitivity to BGL gene expression regulation. However, under the CCR caused by high concentrations of glucose during the cooling of natural compost, the glucose-tolerant BGL genes became upregulated. These results support the hypothesis that BGL-producing microbial communities differentially regulate the expression of glucose-tolerant BGL and non-glucose-tolerant BGL to adapt to changes in cellulose degradation conditions.

## Methods

### Sample collection and physicochemical property analysis

The aerobic composting materials, comprising cattle manure and straw, were obtained from Northeast Agricultural University (Harbin, China). The details of the composting process and the physicochemical properties of straw–cattle manure compost are described by Zang [18]. The lignocellulolytic inoculants (DN-1) (*Phanerochaete chrysosporium*, *Streptomyces griseorubens* C-5, *Bacillus subtilis* W1, *Bacillus methylotrophicus* W8, and *Bacillus amyloliquefaciens* X9. *P. chrysosporium*) were inoculated in the compost, the inoculation amount and strategy are also described by Zang [18]. Composting lasted for 46 days, and piles were turned on day 12 and day 18 according to the temperature. Four time-series samples from the natural and inoculated composts (days 12, 22, 31, and 46) were collected. Samples were immediately frozen in liquid nitrogen and stored at − 80 °C until analysis. Day 22 (thermophilic phase) and day 46 (cooling phase) samples from natural compost and the day 22 (thermophilic phase) and day 31(cooling phase) of inoculated compost were selected and named as TN, CN, TI, CI, respectively, for metatranscriptomics analysis.

### RNA isolation and metatranscriptomic sequencing

RNA was extracted using the RNA PowerSoil Total RNA Isolation Kit (MoBio Laboratories, Inc., Carlsbad, CA USA), the RNA quality after removing potentially contaminating DNA was determined by 1% agarose gels electrophoresis. RNA concentration was confirmed using the Qubit® 3.0 fluorometer (Thermo Fisher Scientific, MA, USA), and RNA integrity and concentration were determined using the RNA Nano 6000 Assay Kit for the Bioanalyzer 2100 system (Agilent Technologies, CA, USA).

A total of 3 μg RNA per sample was used as the input material for RNA preparation. Sequencing libraries were generated using NEBNext® Ultra™ RNA Library Prep Kit for Illumina® (#E7530L, NEB, USA) by following the manufacturer’s instructions, and index codes were added to attribute sequences to each sample.

Briefly, mRNA was purified from total RNA using poly-T oligo-attached magnetic beads for eukaryotes. For prokaryotes, rRNA was removed from total RNA using Ribo-Zero rRNA Removal Kit to purify mRNA. By mixing with the fragmentation buffer, mRNA was fragmented to produce smaller fragments. First-strand cDNA was synthesized using a random hexamer primer, and RNase H. Second-strand cDNA synthesis was subsequently performed using a buffer, dNTPs, DNA polymerase I, and RNase H. The library fragments were purified and resolved with EB buffer, and then a terminal repair, A-tailing, and adapter addition were performed. After size selection and retrieval by AMPure XP beads, the products were used as index PCR templates.

RNA concentration in the library was determined using the Qubit® RNA Assay Kit in Qubit® 3.0 for preliminary quantification and was subsequently diluted to 1 ng/μL. Insert size was assessed using the Agilent Bioanalyzer 2100 system (Agilent Technologies, CA, USA), and the qualified insert size was accurately quantified using the StepOnePlus™ Real-Time PCR system (Thermo Fisher Scientific, MA, USA) (Library valid concentration: > 10 nM). The library was sequenced using Illumina HiSeq™ X TEN.

### Metatranscriptome data analysis

The raw sequence reads were subjected to the filtering of low-quality sequences. The Q30 and GC content of the quality-filtered data was calculated. The remaining quality-filtered sequence reads were assembled de novo into transcripts using Trinity [44] with a minimum assembly length of 200 bp. The transcripts of all four samples were then combined and clustered into unique classes with CD-HIT (cluster database at high identity with tolerance) at 95% identity. After the assembly and clustering of transcripts, the longest sequence of each class was treated as a unigene. The quality of the assembled sequences was assessed by mapping the quality-filtered paired-end reads to the assembled sequences using Bowtie2 with the default parameters implemented in RSEM (RNA-Seq by Expectation Maximization) [45]. The number of reads mapped to each unigene was counted, and the FPKM (fragments per kilobase of transcript sequence per millions of base pairs sequenced) of each gene was calculated based on the length of the gene and the read counts mapped to that gene [46]. The raw sequence reads were submitted to the NCBI Sequence Reads Archive (SRA) as BioProject PRJNA647164. The accession numbers of the read data were SRR1264489, SRR1264490, SRR1264491, and SRR1264492.

### Taxonomic annotation of unigenes and identification of functional genes

A BLAST search (*e*-value < e^−5^) of the unigenes was performed against the NCBI nr database, as each sequence may have multiple hits, the taxonomy was estimated with a custom version of the lowest common ancestor (LCA) to ensure its biological relevance [47]. The CAZy database (https://www.CAZy.org) was used in annotating the functional groups. Glycoside hydrolases (GH) and auxiliary enzyme (AA) families were grouped based on which substrate they act upon, according to the study of Lucia Žifčáková [48] (Additional file 1: Table S1). The transcription regulators (Clrb, XlnR, and creA) were identified and classified based on the BLAST nr database, KEGG database, and EggNOG database with *e*-value < e^−5^.

### DNA extraction and cDNA preparation

Total DNA was extracted from samples using a Hipure Stool DNA Kit (Magen, China), and total RNA was extracted from samples using the RNA PowerSoil Total RNA Isolation Kit (MoBio Laboratories, Inc., Carlsbad, CA USA). cDNA was generated from total RNA using a PrimeScript™ RT reagent Kit with gDNA Eraser (Perfect Real-Time; Takara Bio, Inc.) According to the manufacturer’s instructions. Random hexamers and the RT primer mix were used as primers during strand synthesis. The RT primer mix contains oligo (dT) primers and random 6-mers.

### Construction of DNA library

DNA library targeting GH1 family BGL genes from bacteria of all simples were constructed to analyze the cellulolytic communities in each sample. To identify GH1 BGL, a set of degenerate primers (Additional file 2: Figure S3) was designed based on alignments of nucleotide sequences encoding BGL derived from various known bacteria. Sequences were analyzed using the BLAST program and the Genbank databases (https://www.ncbi.nlm.nih.gov/). Multiple sequence alignments were conducted with the Clustal W algorithm, and the alignment and positions of primers are shown in Additional file 2: Figure S3. PCR reactions were conducted in triplicate (50 μL of the reaction mixture containing 25 μL 2 × Taq Master Mix [novoprotein], 0.4 μmol/L of each primer, 1 μL of DNA templates, and 23.2 μL ddH_2_O). Amplification was conducted as follows: initial denaturation at 95 °C for 5 min; 30 cycles of 95 °C for 30 s; annealing for 30 s at 50.0 °C and 72 °C for 90 s; and final elongation at 72 °C for 10 min. Pooled PCR products were visualized on a 1% agarose gel and then purified using the Gel Extraction Kit (Omega). The purified products were ligated to pMD™ 18-T Vector (Takara, USA) and transformed into *E. coli* DH5α Competent Cells (Takara, USA) by heat shock. The recombinant plasmids were placed in solid Luria–Bertani (LB) medium containing X-gal, IPTG, and ampicillin (AMP +) and cultured for 12 h at 37 °C. Meanwhile, single white colonies were inoculated in liquid LB medium containing AMP + and cultured for 12 h at 37 °C. After the bacterial liquid was used as the PCR template to amplify the target gene, the amplified products were detected by agarose gel electrophoresis. Positive clones containing the gene fragment were sent to Beijing Huada Gene Company (Beijing, PR China) for sequencing.

### Real-time quantitative PCR of BGL genes and data analysis

CFD-3120 real-time PCR system (Bio-Rad) was used to conduct qPCR. DNA and RNA were used as the template to quantify the BGL genes belonging to the GH1 family, and the fluorescent dye SYBR Green was used for performing quantitative analysis of the functional gene. Each reaction was conducted in a 20 μL system, including 10 μL of NovoStart® SYBR qPCR SuperMix Plus (2 × , novoprotein, China). The primers used for qPCR in this study are provided separately (Additional file 1: Table S2). Amplification was performed after 60 s of denaturation at 95 °C, and 40 cycles of PCR were performed at 95 °C for 20 s and 60 °C for 60 s. Melting curve analysis was used to confirm product specificity. All assays were repeated 3 times, and no signal was observed in negative controls.

The ratio of cDNA copy number and DNA copy number was defined to determine the TE of BGL genes. The TE growth rate [Eq. (1)] was used to describe the trend of individual BGL genes in transcriptional regulation during the composting process. The average value of the TE growth rate was used to describe the trend in BGL between the two groups with or without conserved residues Trp168 and Leu173, respectively. Equation (1) shows a mathematical model of TE growth rate of individual BGL genes in *t*2 phase compared with *t*1 phase during composting:

1 $$ {\text{Rate}} = \left( {{\text{TE}}_{t2} - {\text{TE}}_{t1} } \right)/{\text{TE}}_{t1} , $$

where TE_*t*1_ represents the transcription efficiency of BGL in the *t*1 phase, and TE_*t*2_ is the transcription efficiency of BGL in the *t*2 phase.

### Statistical analysis

Data were analyzed using the SPSS 13.0 program for windows. One-way ANOVA with repeated measures was used to test differences in the measured parameters during composting, and the post hoc Tukey test was used to further investigate the differences. *P* < 0.05 was considered to be statistically significant. DEGseq, an R package, was used to identify differentially expressed genes. GraphPad Prism 8 (GraphPad Software, Inc., USA) was used for data analysis and drawing data charts.

## Supplementary information

**Additional file 1: Table S1.** Functional classification of glycosyl hydrolases and auxiliary used in this paper based on their characterized catalytic activities according to CAZy (https://www.cazy.org/). **Table S2.** Primers for β-glucosidase genes used for qPCR. The primers were designed by Primer-BLAST in NCBI. Primer pairs are specific to amplified sequences as no other targets were found in selected database: RefSeq Representative Genome Database (Organism limited to Bacteria, Fungi, Cow, Zea mays).

**Additional file 2: Figure S1.** Phylogenetic analysis based on the partial amino acid sequences of GH1 family β-glucosidase genes selected from representative sequences used to design qPCR primers and their relationship with the reference sequences retrieved from Genbank. The tree was constructed using the neighbor-joining method. **Figure S2.** Transcription efficiency of GH1 family β-glucosidase genes from bacteria in the natural composting and the inoculated composting. **Figure S3.** Nucleotide sequence alignment of GH1 family BGL genes and primers used for this study.

## Data Availability

All data generated or analyzed during this study are included in this published article.

## Abbreviations

GH: Glycoside hydrolases
AA: Auxiliary activities
CAZy: Carbohydrate‑active enzymes
BGL: β-Glucosidase
pNPG: *p*-Nitrophenyl-β-D-glucopyranoside
CCR: Carbon catabolite repression
CMC: Carboxymethyl cellulose
FPKM: Fragments per kilobase of transcript sequence per millions of base pairs sequenced
SRA: Sequence reads archive
LCA: Lowest common ancestor
IPTG: Isopropyl-β-thiogalactopyranoside
