# Supplementary material for: β-Glucosidase genes differentially expressed during composting
Source: Biotechnol Biofuels. 2020 Oct 19;13:174. doi: 10.1186/s13068-020-01813-w (PMC7570026; doi:10.1186/s13068-020-01813-w)
Supplement: Supplementary file 1 — Additional file 1: Table S1. Functional classification of glycosyl hydrolases and auxiliary used in this paper based on their characterized catalytic activities according to CAZy (https://www.cazy.org/). Table S2. Primers for β-glucosidase genes used for qPCR. The primers were designed by Primer-BLAST in NCBI. Primer pairs are specific to amplified sequences as no other targets were found in selected database: RefSeq Representative Genome Database (Organism limited to Bacteria, Fungi, Cow, Zea mays). [file 13068_2020_1813_MOESM1_ESM.docx]

**Table S1:** Functional classification of glycosyl hydrolases and auxiliary used in this paper based on their characterized catalytic activities according to CAZy (http://www.cazy.org/)

| Group | Target | GH families |
| --- | --- | --- |
| Starch/glycogen | Storage compounds | GH13 (amylase/α-glucosidase/trehalase), GH14 (amylase), GH15 (glucoamylase/glucodextranase), GH31 (α-glucosidase), GH57 (amylase), GH77 (amylomaltase), GH119 (amylase) |
| Trehalose | Storage compounds | GH37 (trehalase), GH65 (trehalase) |
| Cellulose | Plant cell wall | GH5_1, GH5_2, GH5_4, GH5_5, GH5_25, GH5_26, GH5_38, GH5_39, GH5_46 (endocellulase), GH6(exocellulase/endocellulase), GH7 (exocellulase/endocellulase), GH8 (endocellulase/endoxylanase), GH9 (exocellulase/endocellulase/endoxylanase/β-glucosidase), GH12 (endocellulase/endoxylanase), GH44 (endocellulase/endoxylanase), GH45 (endocellulase), GH48 (exocellulase/endocellulase/chitinase), GH74 (endocellulase), AA9 (lytic polysaccharide monooxygenase) |
| Lignin | Plant cell wall | AA1 (laccase), AA2 (peroxidase), AA3 (oxidase), AA4 (oxidase), AA5 (oxidase), AA6 (oxidase), AA12 (oxidase) |

**Table S2** Primers for β-glucosidase genes used for qPCR. The primers were designed by Primer-BLAST in NCBI. Primer pairs are specific to amplified sequences as no other targets were found in selected database: RefSeq Representative Genome Database (Organism limited to Bacteria, Fungi, Cow, Zea mays).

| Primer | Forward Sequence (5'-3') | Reverse Sequence (5'-3') | Accession number |
| --- | --- | --- | --- |
| GH1B-3-55 | GGCGATGCGATGTTCAATGG | CAATCTCCATGTCGCGATGC | MN786821 |
| GH1B-b4-18 | TGGCAACGCACAACTGTTAC | ATGTCGTCCGACTTGTTGGT | MT001429 |
| GH1B-7-4-17 | GAATCGCACAGATGCGTAGG | TGCGTACCCAACTAATCGCC | MN939771 |
| GH1B-12-33 | CACTGAATCTGACGAATGCGG | CATCACATCAATGCGGCGTG | MT542131 |
| GH1B-13-55 | CATTCATCCGCGACCACATC | AGCGACCACACGAAGTAGC | MT001431 |
| GH1B-13-12 | CAAGCTGCACCAAACCTACG | AGGTGGCGTTCATAGTAGCG | MT001430 |
| GH1B-7-41 | GCGCACTTGATGTCTCATGC | ACGCGCATCCATTCTCTGTA | MN792777 |
| GH1B-13-33 | ACACAGGCTATGATTCCCGC | GCCGGAGTATGGCTGAACAT | MT001432 |
| GH1B-10-4-50 | TCCCACAACCTTCTGCTGTC | CCACCTGTTATGGATGCCGT | MN792779 |
| GH1B-14-31 | GGTGATCTGGTGGCAACACT | ACCAGATCGCTTCCTGTTCG | MN958075 |
| GH1B-b2-20 | TAGGTACTACATTTTCGTGCTCACA | CGTGCTGTTGCATGTAATTTTCAAT | MN786817 |
| GH1B-b4-26 | TTTCAAGGGCTCTTATCCGAAGA | CAGCGTAAATATTGAGACCCACG | MN786822 |
| GH1B-b4-37 | TCCCGTCTTACTCACAGCAAC | AACCTCGACCGTAGATTGGG | MN786809 |
| GH1B-3-66 | ATCCGAAGCAGCGCATTACT | GCGTCATGCACACATCCATC | MN958076 |
| GH1B-6-19 | CAGGTTTGCAGCTTCCGTTG | GCGTAGCCAATGCTCAGTTC | MN958077 |
| GH1B-13-2-6 | CCATGGGAGACTATGCGGAG | GGAATCGACACCCACGGATT | MN812765 |
| GH1B-b4-61 | CTCCCGTCTTACTCACAGCAAC | GGATAACCTCGACCGTAGATTGCG | MN786813 |
| GH1B-14-21 | GGACGCGGTTATCCTGAAGA | TTGTAAAGCCCACGCGGATA | MN939772 |
