# Supplementary material for: β-Glucosidase genes differentially expressed during composting
Source: Biotechnol Biofuels. 2020 Oct 19;13:174. doi: 10.1186/s13068-020-01813-w (PMC7570026; doi:10.1186/s13068-020-01813-w)
Supplement: Supplementary file 2 — Additional file 2: Figure S1. Phylogenetic analysis based on the partial amino acid sequences of GH1 family β-glucosidase genes selected from representative sequences used to design qPCR primers and their relationship with the reference sequences retrieved from Genbank. The tree was constructed using the neighbor-joining method. Figure S2. Transcription efficiency of GH1 family β-glucosidase genes from bacteria in the natural composting and the inoculated composting. Figure S3. Nucleotide sequence alignment of GH1 family BGL genes and primers used for this study. [file 13068_2020_1813_MOESM2_ESM.docx]

**Fig. S1** Phylogenetic analysis based on the partial amino acid sequences of GH1 family β-glucosidase genes selected from representative sequences used to design qPCR primers and their relationship with the reference sequences retrieved from Genbank. The tree was constructed using the neighbor-joining method.

**Fig. S2** Transcription efficiency of GH1family β-glucosidase genes from bacteria in the natural composting and the inoculated composting.


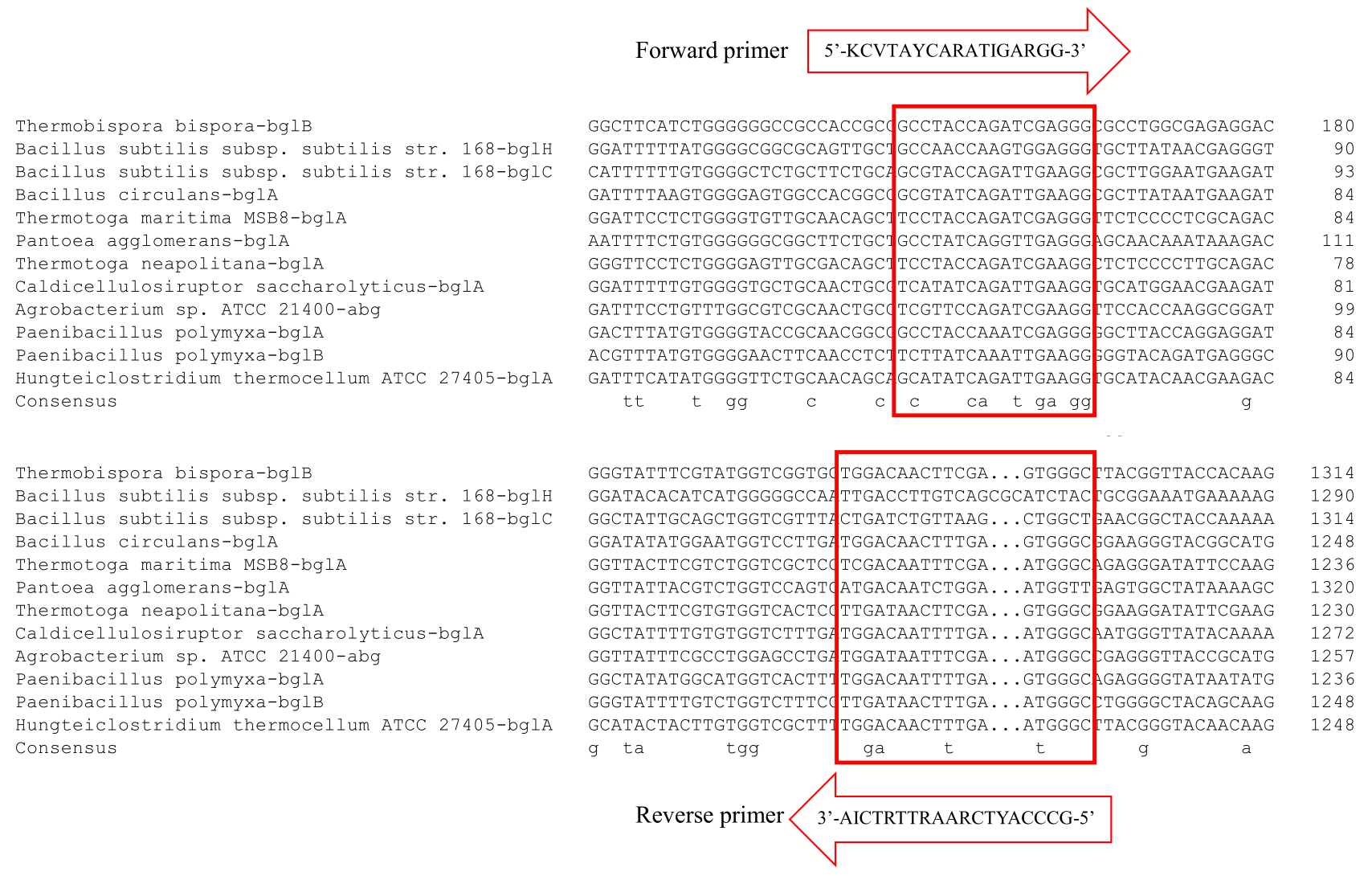


**Fig. S3** Nucleotide sequence alignment of GH1 family BGL genes and primers used for this study. The nucleotide sequence alignment can be found at GenPept and GenBank: *Thermobispora bispora* -bglB (database accession number P38645), B*acillus subtilis subsp. subtilis str.* 168-bglH (P40740), *Bacillus subtilis subsp. subtilis str.* 168-bglC (P42403), *Bacillus circulans*-bglA (Q03506), *Thermotoga maritima* MSB8-bglA (Q08638), *Pantoea agglomerans*-bglA (Q59437), *Thermotoga neapolitana*-bglA (Z97212), *Caldicellulosiruptor saccharolyticus-*bglA (P10482), *Agrobacterium sp.* ATCC 21400-abg (P12614), *Paenibacillus polymyxa*-bglA (P22073), *Paenibacillus polymyxa*-bglB (P22505), *Hungteiclostridium thermocellum* ATCC 27405-bglA (P26208).
